# Early and Regular Bronchoscopy Examination on Effect of Diagnosis and Prognosis for Patients With Tracheobronchial Tuberculosis

**DOI:** 10.3389/fmed.2022.825736

**Published:** 2022-02-15

**Authors:** Tingting Hu, Yishi Li, Xiaohui Wang, Yan Chen, Xiao Nie, Rongjuan Zhuang, Ying Li, Shuliang Guo

**Affiliations:** ^1^Department of Respiratory and Critical Care Medicine, The First Affiliated Hospital of Chongqing Medical University, Chongqing, China; ^2^Department of Respiratory and Critical Care Medicine, The First Affiliated Hospital of Chongqing Medical and Pharmaceutical College, Chongqing, China; ^3^Department of Respiratory and Critical Care Medicine, Affiliated Hospital of North Sichuan Medical College, Nanchong, China

**Keywords:** tracheobronchial tuberculosis, bronchoscopy, early diagnosis, treatment, tracheobronchial stenosis

## Abstract

**Background:**

Bronchoscopy is the main method for the diagnosis of tracheobronchial tuberculosis (TBTB). However, it is not well-used in patients with pulmonary tuberculosis (PTB), leading to misdiagnosis. The aim of this study is to verify the value and feasibility of bronchoscopy for an early diagnosis and treatment of TBTB.

**Materials and Methods:**

A prospective observational study was performed in patients with active PTB. The ratios of TBTB and tracheobronchial stenosis were analyzed with propensity score matching (PSM) for baseline characteristics, and a Cox regression model was further employed to adjust for residual confounding factors.

**Results:**

A total of 656 patients with active PTB were enrolled in the study that included 307 patients in the active group and 349 patients in the non-active group. The ratio of TBTB was significantly higher in the active group than that in the non-active group [hazard ratio (*HR*), 2.31; 95% *CI*, 1.70–3.14; *p* < 0.001]. With PSM, the proportion of tracheobronchial stenosis in the non-active group was significantly higher than that in the active group (*HR*, 1.84; 95% *CI*, 1.15–2.95; *p* = 0.011). Moreover, the number of patients with moderate to severe stenosis were significantly higher than that in the active group (*HR*, 4.13; 95% *CI*, 2.25–7.63; *p* < 0.001). Similar results were obtained with multivariate analysis. With 12 months of treatment, both therapeutic effective rate (84.7 vs. 68.2%; *p* = 0.009) and improvement rate of non-fibrotic tracheobronchial stenosis (79.1 vs. 47.4%; *p* = 0.022) were higher in the active group than that in the non-active group.

**Conclusion:**

Active and regular bronchoscopy is conducive to early diagnosis of TBTB, combined with prompt anti-tuberculosis therapy, greatly reducing the occurrence of tracheobronchial stenosis and improving prognosis.

## Introduction

Pulmonary tuberculosis (PTB) is a chronic respiratory infectious disease that seriously endangers human health. It is caused by *Mycobacterium tuberculosis* and is one of the most common infectious diseases in China. According to the report released by the WHO in 2020, there were about 10 million new cases of tuberculosis worldwide in 2019, of which 1.5 million died from it. China is the worst-hit region for tuberculosis, with new cases accounting for about 8.4% each year. The tuberculosis epidemic imposes a severe economic burden on the government and the families of patients (1).

Tracheobronchial tuberculosis (TBTB) is a special type of tuberculosis occurring in the mucosa, submucosa, and outer membrane of trachea and bronchus and often results in tracheobronchial stenosis, which can lead to repeated hospitalizations due to breathing difficulties and lung infections (2, 3). The available data show that about 10–40% of patients with PTB are complicated with TBTB (4–6). With the popularity of bronchoscopy, the incidence of TBTB may actually be up to 54.3% (7). Therefore, it is imperative to raise awareness of TBTB and develop effective early diagnosis and treatment measures.

Bronchoscopy is the main method to diagnose TBTB (8). In a multicenter study, Su et al. performed bronchoscopy on 1,441 patients with PTB and found that 23.9% of them were diagnosed with TBTB (5). Um et al. suggested early bronchoscopy examination was beneficial to prompt the diagnosis of TBTB (3). These published studies highlight the central role of bronchoscopy in diagnosis of TBTB. However, it remains unclear whether early and regular bronchoscopy examination has an impact on the prevalence and prognosis of TBTB in patients with PTB. Therefore, the aim of this study is to verify the value and feasibility of early diagnosis of TBTB by performing bronchoscopy actively in patients with PTB.

## Materials and Methods

### Law and Ethics

Prospective registration and reporting of clinical trials: adopted and implemented in accordance with ICMJE and Declaration of Helsinki (registration number: ChiCTR1900023525). The study was approved by the Medical Ethics Committee of the First Affiliated Hospital of Chongqing Medical University.

### Inclusion and Exclusion Criteria

This study is a prospective observational study conducted in the First Affiliated Hospital of Chongqing Medical University in China in 2019. Recruitment took place in this hospital from July 2019 to July 2020. The inclusion criteria of subjects were: (1) Patients with the initial diagnosis of active PTB, confirmed by a positive acid-fast bacilli smear or culture of *Mycobacterium tuberculosis* or histopathological evidence; (2) patients who were conscious and cooperative; and (3) patients who voluntarily took the study and signed an informed consent form. The exclusion criteria of subjects were: (1) there were contraindications to bronchoscopy examination that cannot be corrected within a short period of time; (2) patients who were combined with the cardiovascular, hepatic, hematopoietic system, or other serious diseases that can threaten lives; and (3) patients who were participating in other clinical trials. Once PTB was diagnosed, all patients would receive a standard four-drug combination anti-tuberculosis treatment with Rifampicin, Isoniazid, Pyrazinamide, and Ethambutol. For patients with intolerance or drug resistance, clinicians would adjust the treatment regimen as appropriate.

### Study Design

**Figure 1:**
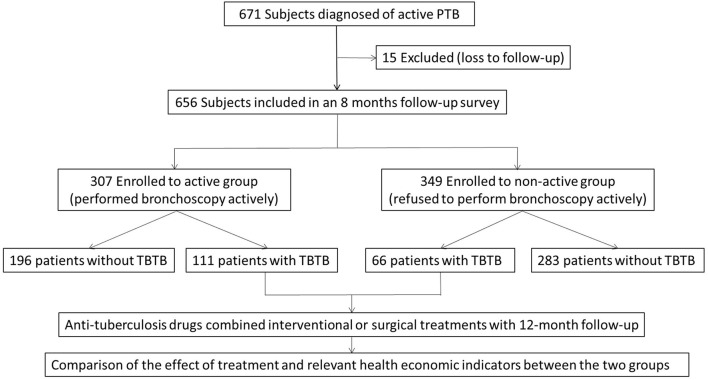
A flow diagram of the study.

Patients performed with bronchoscopy actively and regularly were enrolled to the active group. At the 2nd, 4th, 6th, and 8th months after initial diagnosis of PTB, bronchoscopy was performed to confirm the presence of luminal changes. Patients who did not want to undergo regular bronchoscopy examination until visiting for symptoms (such as, shortness of breath, dyspnea, and other discomforts) were enrolled to the non-active group. The routine follow-up was the same as that of active group. Once TBTB was found, the specimen would be obtained in time to clarify the nature of new lesions, and bronchoscopic findings (the subtype of TBTB and degree of tracheobronchial stenosis) were recorded. All subjects in the non-active group confirmed the presence or absence of TBTB by bronchoscopy at the last month of follow-up. After TBTB was diagnosed, anti-tuberculosis drugs combined with interventional (such as, thermal ablation, cryotherapy, and balloon dilation) or surgical treatment were performed according to the condition of patients. The therapeutic effect would be observed with 12-month follow-up. A flow diagram of the study is shown in Figure 1.

### Outcomes

Primary outcomes were the ratio of patients with TBTB and degree of tracheobronchial stenosis due to TBTB. The diagnostic criteria for TBTB is: typical tracheobronchial visible lesions under bronchoscopy, such as mucosal hyperemia, edema, ulcer, necrosis, granuloma, stenosis, and lymph fistula, and either positive etiological examinations or histopathologic diagnosis. The sampling techniques for etiology included bronchial washing, brushing, and endobronchial biopsy (6). China is the worst-hit region for tuberculosis. Respiratory physicians in China have accumulated a lot of experience in the diagnosis and treatment of tuberculosis. They updated and revised the classification of TBTB according to the study by Chung et al., forming a new expert consensus in line with Chinese patients. In light of features of luminal lesions, TBTB was classified as the following subtypes: inflammatory infiltration, ulcer necrosis, granulation hyperplasia, cicatricial stenosis, tracheobronchial malacia, and lymph fistula. Bronchoscopic manifestations of each subtype are shown in the supplement (6, 9). The severity of tracheobronchial stenosis was graded as non-stenosis or a 25, 50, 75, and 90% decrease or complete obstruction according to the cross-sectional area of the airway (10).

Secondary outcomes were the therapeutic effective rate, prognosis of airway stenosis, times of interventional treatment, total costs of therapy, and length of stay in the hospital. Overcomes of treatment included improvement, no change, and aggravation. The criteria for bronchoscopic improvement were regression of hyperemia and edema, clearing of granulation tissue, cessation of exuding caseous material, and reduction of tracheobronchial stenosis. Aggravation was defined as the last bronchoscopic finding worsening. Other cases were defined as no change. The therapeutic effective rate was defined as the percentage of TBTB who responded to the treatment out of the total number of TBTB. Serial bronchoscopic findings were comparatively analyzed every 3 months until the resolution of TBTB was observed. Similarly, the follow-up of airway stenosis was assessed as aggravation, no change, or improvement.

### Statistical Analysis

Summary statistics were presented as percentages in the case of categorical variables and as means with SDs in the case of continuous variables. The baseline characteristics of active group and non-active group were evaluated by Pearson's chi-square test or Fisher's exact test for categorical variables and Wilcoxon rank-sum test for continuous variables. Kaplan–Meier survival curve and Log-Rank were used to assess the relationship between active bronchoscopy examination and TBTB's proportion. Hazard ratios (*HR*s) and 95% *CI* were calculated by using a Cox proportional hazard model, which examined the relationship between active bronchoscopy examination and the relative risk of TBTB's proportion. Furthermore, a logistic regression-based multivariate analysis was used to adjust for residual imbalance by including parameters with *p* < 0.05 and potential confounders judged by clinical expertise. Finally, the Mann–Whitney *U*-test was used for analysis of efficacy and the healthcare related economic indicators of treatment.

The propensity score was used to account for baseline differences in the probability to receive or not an active bronchoscopy examination based on observed covariates. Confounders, such as age, gender, residence, education, body mass index, course of PTB, smoking history, diabetes, other respiratory diseases, cough grade, sputum grade, cavity in chest image, course of anti-tuberculosis treatment, medicare, and income were taken into propensity score matching (PSM). We used a 1:1 matching algorithm between the active group and non-active group within a caliper of 0.2 SD of the logit of the propensity score. Absolute standardized differences and values of *p* were determined for all baseline variables before and after matching to evaluate the imbalance. The logistic regression was then performed on matched cohort, adjusting for the variables that remained unbalanced between the two groups.

Statistical analysis was performed with SPSS version 26 (SPSS Inc., Chicago, IL, USA). All analyses were carried out as two-tailed tests. A *p* < 0.05 was considered significant.

## Results

### Characteristics of the Study Population

A total of 656 subjects were admitted to this study. Of them, 307 patients with PTB underwent active and regular bronchoscopy examination, which were defined as the active group. Moreover, 349 patients did not undergo regular bronchoscopy examination until visiting for symptoms, which were defined as the non-active group. Characteristics of the overall population are presented in Table 1. In general, patients in the active group were older and had lower level of education.

**Table 1:** Baseline characteristics of the patients with PTB before and after propensity score matching after an 8-month of following-up.

**Characteristics**	**Overall cohort**				**Matched cohort**			
	**Active group**	**Non-active group**	**ASD (%)**	***P*-value**	**Active group**	**Non-active group**	**ASD (%)**	***P*-value**
	**(*N* = 307)**	**(*N* = 349)**			**(*N* = 220)**	**(*N* = 220)**		
Age, Mean ± SD	42.3 ± 17.8	38.4 ± 16.5	22.1	0.006	39.7 ± 17.3	40.0 ± 17.0	1.7	0.688
Female sex (%)	136 (44.3)	162 (46.4)	4.3	0.587	103 (46.8)	101 (45.9)	1.8	0.848
Live in the rural (%)	113 (36.8)	118 (33.8)	6.2	0.423	80 (36.4)	79 (35.9)	0.9	0.921
Education below university (%)	232 (75.6)	218 (62.5)	30.5	<0.001	156 (70.9)	156 (70.9)	0.0	1.000
BMI, Mean ± SD	21.4 ± 3.5	21.5 ± 3.3	2.4	0.657	21.4 ± 3.5	21.4 ± 3.5	0.1	0.830
Course of PTB ≥ 6 months (%)	53 (17.3)	52 (14.9)	6.2	0.410	34 (15.5)	33 (15.0)	1.2	0.894
Current smoker (%)	78 (25.4)	54 (15.5)	22.8	0.002	37 (16.8)	43 (19.5)	6.3	0.458
Combined with diabetes (%)	18 (5.9)	34 (9.7)	16.5	0.067	17 (7.7)	19 (8.6)	3.9	0.728
Combined with other respiratory diseases (%)	27 (8.8)	21(6.0)	9.8	0.173	11 (5.0)	13 (5.9)	3.2	0.675
Cough (%)				0.611				0.843
None	65 (21.2)	74 (21.2)			44 (20.0)	46 (20.9)		
Mild	172 (56.0)	193 (55.3)	1.5		136 (61.8)	129 (58.6)	6.4	
Moderate	57 (18.6)	43 (12.3)	16.0		29 (13.2)	34 (15.5)	5.8	
Severe	13 (4.2)	39 (11.2)	34.4		11 (5.0)	11 (5.0)	0.0	
Sputum (%)				0.593				0.570
None	90 (29.2)	99 (28.4)			66 (29.1)	64 (29.1)		
Little	174 (56.7)	197 (56.4)	0.5		129 (58.6)	125 (56.8)	3.7	
Medium	37 (12.1)	33 (9.5)	8.0		21 (9.5)	25 (11.4)	5.6	
Large	6 (2.0)	20 (5.7)	27.2		4 (1.8)	6 (2.7)	6.6	
Cavity in chest image (%)	67 (21.8)	89 (25.5)	8.9	0.270	50 (2.27)	55 (2.5)	5.5	0.576
Course of anti-tuberculosis treatment (month), Mean ± SD	2.4 ± 3.3	2.6 ± 3.7	6.7	0.191	2.5 ± 3.5	2.6 ± 3.9	2.7	
Medicare (%)				0.976				0.880
Self-supporting	30 (9.8)	33 (9.4)			19 (8.6)	21 (9.6)		
Rural medical insurance	78 (25.4)	91 (26.1)	1.5		58 (26.4)	54 (24.5)	4.2	
Urban medical insurance	199 (64.8)	225 (64.5)	0.7		143 (65.0)	145 (65.9)	1.9	
Income (%)				0.215				0.130
Low	58 (18.9)	46 (13.3)			42 (19.0)	33 (15.0)		
Middle	185 (60.3)	229 (65.5)	10.9		133 (60.5)	131 (59.5)	1.9	
High	64 (20.8)	74 (21.2)	0.9		45 (20.5)	56 (25.5)	12.3	

*PTB, pulmonary tuberculosis; ASD, absolute standardized differences; SD, standard deviation. BMI, the body-mass index, is the weight in kilograms divided by the square of the height in meters*.

### Primary Outcomes

**Figure 2:**
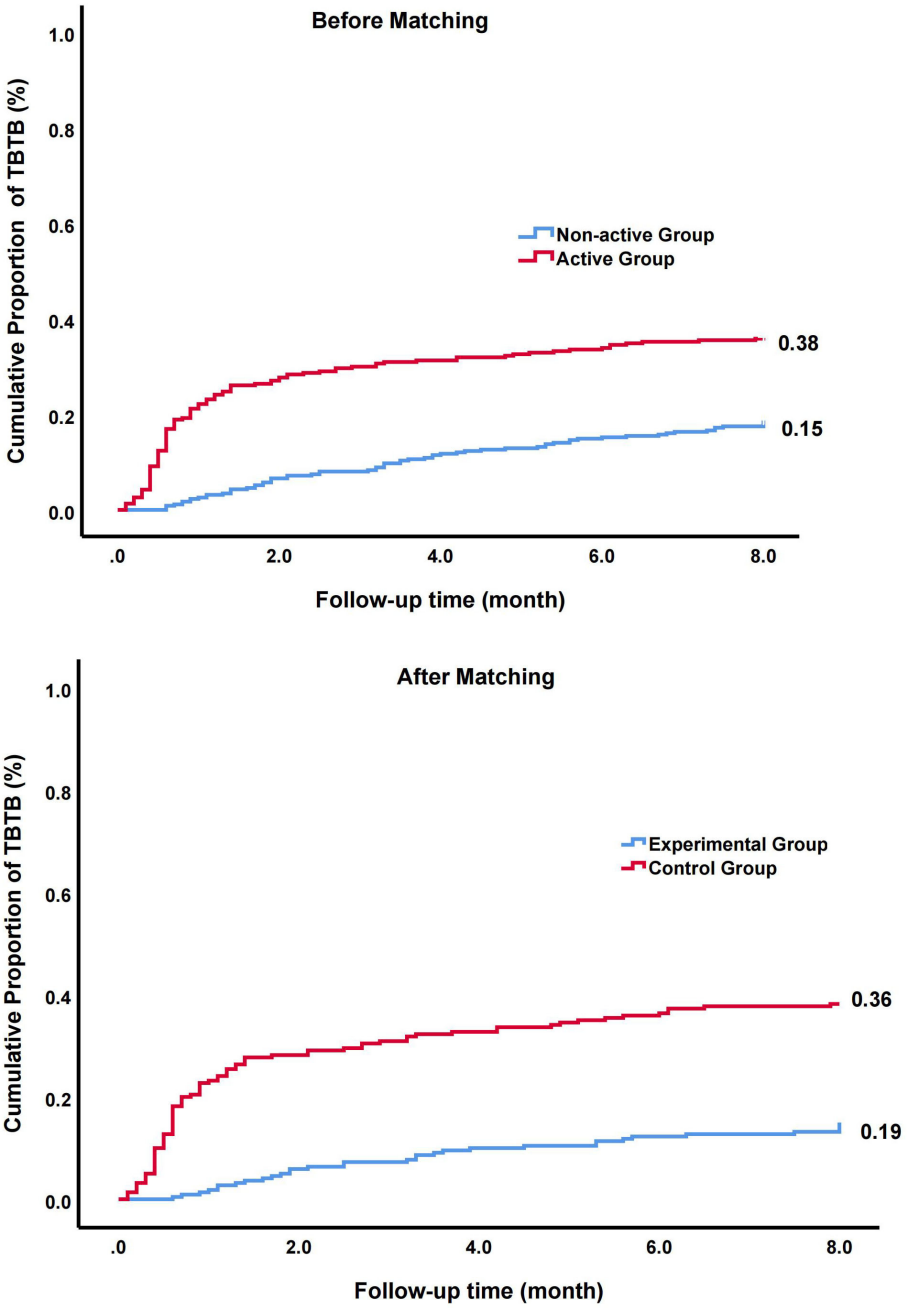
The cumulative proportion and hazard ratio (*HR*) of patients with tracheobronchial tuberculosis (TBTB) in overall cohort and matched cohort.

The ratio of TBTB was 0.38 in the active group and 0.15 in the non-active group (Figure 2). A univariate analysis showed that the ratio of patients with TBTB in the active group was significantly higher than that in the non-active group (*HR*, 2.31; 95% *CI*, 1.70–3.14; *p* < 0.001). Similar results were obtained when multivariate analysis was further used to control confounders (*HR*, 2.40; 95% *CI*, 1.76–3.28; *p* < 0.001) (Table 2).

**Table 2:** Hazard ratio (*HR*) and 95% *CI* were calculated by using a Cox proportional hazard model.

	**Overall cohort**		**Matched cohort**	
	**Univariate**	**Multivariate**	**Univariate**	**Multivariate**
	**analysis**	**analysis**	**analysis**	**analysis**
HR (95% CI)	2.31 (1.70–3.14)	2.40 (1.76–3.28)	3.14 (2.10–4.70)	3.13 (2.09–4.68)
*P*-value	<0.001	<0.001	<0.001	<0.001

*HR, hazard ratio; CI, confidence interval*.

*A logistic regression-based multivariate analysis was used to adjust for residual imbalance, such as age, gender, education, and diabetes, in the pre-match and post-match cohorts*.

Probability scores were next calculated. The imbalance between the two groups were significantly reduced after PSM (Table 1). In matched cohort, a univariate analysis showed that the ratio of patients with TBTB in the active group was significantly higher than that of non-active group (*HR*, 3.14; 95% *CI*, 2.10–4.70; *p* < 0.001). To further control residual confounders, the multivariate analysis was used. The results were consistent with above (*HR*, 3.13; 95% *CI*, 2.09–4.68; *p* < 0.001) (Table 2).

Bronchoscopic characteristics in matched cohort are shown in Table 3. The proportion of cicatricial stenosis (32.1 vs. 63.6%, *p* < 0.001) and tracheobronchial malacia (0.0 vs. 9.1%, *p* < 0.001) in the active group was lower than that in the non-active group. Univariate analysis showed that the ratio of tracheobronchial stenosis in the non-active group was significantly higher than that in the active group (*HR*, 1.84; 95% *CI*, 1.15–2.95; *p* = 0.011). The number of patients with moderate-severe stenosis (stenotic degree ≥50%) were significantly higher than that in the active group (*HR*, 4.13; 95% *CI*, 2.25–7.63; *p* < 0.001). After the multivariate analysis was performed, the results were consistent with the above findings (Table 4).

**Table 3:** Bronchoscopic characteristics of the patients with tracheobronchial tuberculosis (TBTB).

	**Overall cohort**			**Matched cohort**		
	**Active group**	**Non-active group**	***P*-value**	**Active group**	**Non-active group**	***P*-value**
	**(*N* = 111)**	**(*N* = 66)**		**(*N* = 84)**	**(*N* = 33)**	
Subtype (%)			<0.001			0.001
Inflammatory infiltration	28 (25.2)	4 (6.1)		25 (29.8)	2 (6.1)	
Ulcer necrosis	32 (28.8)	9 (13.6)		24 (28.6)	6 (18.2)	
Granulation hyperplasia	7 (6.3)	2 (3.0)		4 (4.8)	0 (0.0)	
Cicatricial stenosis	37 (33.3)	41 (62.1)		27 (32.1)	21 (63.6)	
Tracheobronchial malacia	2 (1.8)	7 (10.6)		0 (0.0)	3 (9.1)	
Lymph fistula	5 (4.5)	3 (4.5)		4 (4.8)	1 (3.0)	
Stenotic degree (%)			0.002			0.001
None	31 (27.9)	6 (9.1)		29(34.5)	2 (6.1)	
≤ 25%	33 (29.7)	12 (18.2)		21 (25.0)	7 (21.2)	
26–50%	21 (18.9)	14 (21.2)		16 (19.0)	6 (18.2)	
51–75%	11 (9.9)	11 (16.7)		9 (10.7)	6 (18.2)	
76–90%	4 (3.6)	10 (15.2)		1 (1.2)	6 (18.2)	
≥91%	11 (9.9)	13 (19.7)		8 (9.5)	6 (18.2)	
Stenotic degree>50%	26 (23.4)	34 (51.5)	<0.001	18 (21.4)	18 (54.5)	<0.001

**Table 4:** Hazard ratio of TBTB with tracheobronchial stenosis and stenotic degree ≥50% in matched cohort.

	**Univariate analysis**			**Multivariate analysis**		
	**HR**	**95% CI**	***P*-value**	**HR**	**95% CI**	***P*-value**
TBTB with tracheobronchial stenosis	1.84	1.15–2.95	0.011	1.96	1.19–3.22	0.008
TBTB with stenotic degree ≥50%	4.13	2.25–7.63	<0.001	4.12	2.19–7.75	<0.001

*HR, hazard ratio; CI, confidence interval*.

### Secondary Outcomes

**Figure 3:**
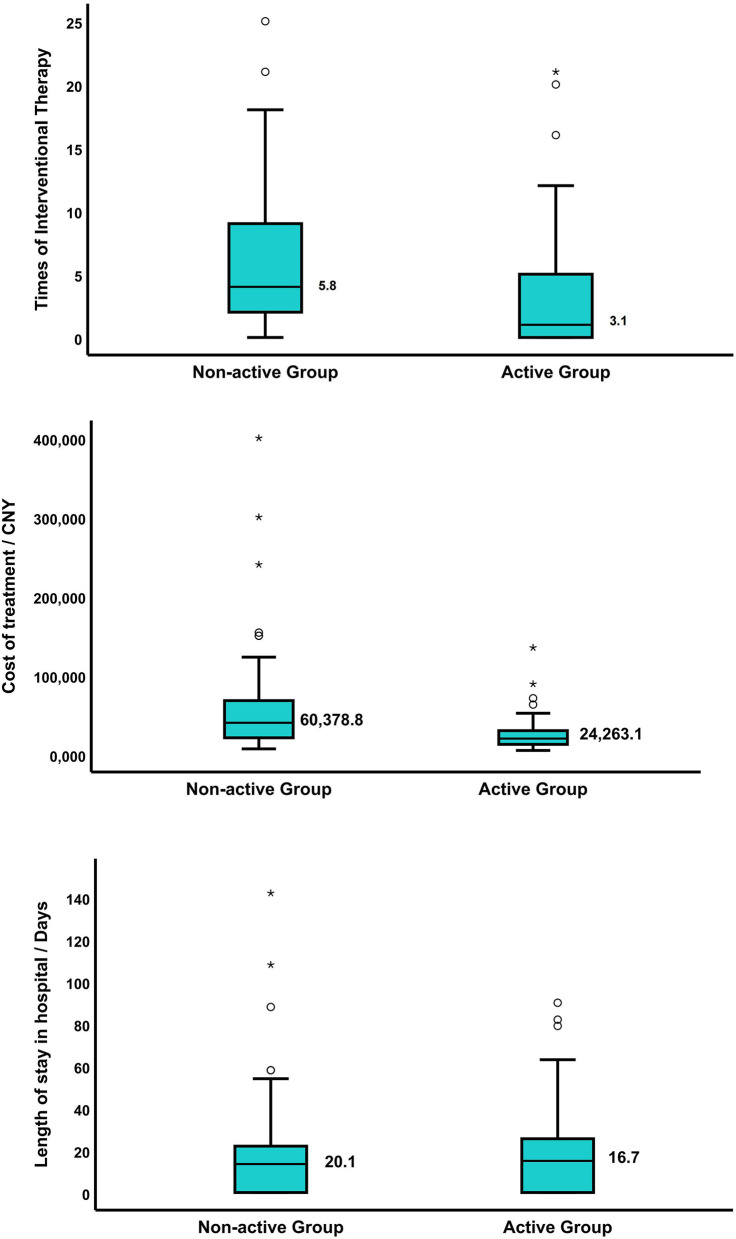
**(A)** The times of interventional therapy were 5.8 in the non-active group and 3.1 in the active group, *p* < 0.001; **(B)** the total cost of therapy in patients with TBTB in the non-active group was 60,378.8 CNY, and that of the active group was 24,263.1 CNY, *p* < 0.001; **(C)** the length of stay in the hospital of patients with TBTB was 20.1 days in the non-active group and 16.7 days in the active group, *p* = 0.844. The symbol ^*^ means a significant outlier, indicating that the value is outside Q1−3IQR or Q3+3IQR.

After 12 months of treatment, an improved bronchoscopic finding of TBTB was observed in 84.7% in the active group and 68.2% in the non-active group (*p* = 0.009) (Table 5). The rate of improvement in stenosis without fibrosis was 79.1% in the active group and 47.4% in the non-active group (*p* = 0.022). Notably, 16.3% patients with non-fibrosis stenosis progressed to fibrotic stenosis in the active group vs. 31.6% in the non-active group. For patients with fibrotic stenosis, no significant difference was detected in therapeutic effect between the two groups (Table 6). Otherwise, times of interventional therapy (5.8 vs. 3.1; *p* < 0.001) and total costs of therapy (60,378.8 vs. 24,263.1 CNY; *p* < 0.001) were significantly higher in the non-active group than that in the active group. The improvement in stenosis with fibrosis and length of stay in the hospital did not significantly differ (Figure 3).

**Table 5:** Prognosis between two groups after a 12-month treatment.

	**Improvement**	**No change**	**Aggravation**	***P*-value**
Active group (%)	94 (84.7)	14 (12.6)	3 (2.7)	0.009
Non-active group (%)	45 (68.2)	16 (24.2)	5 (7.6)	

**Table 6:** Change in grade of tracheobronchial stenosis after a 12-month treatment.

	**Stenosis without fibrosis**				**Stenosis with fibrosis**			
	**Improvement**	**No change**	**Progression to fibrosis**	***P*-value**	**Improvement**	**No change**	**Aggravation**	***P*-value**
Active group (%)	34 (79.1)	2 (4.7)	7 (16.3)	0.022	30 (81.1)	7 (18.9)	0 (0.0)	0.360
Non-active group (%)	9 (47.4)	4 (21.1)	6 (31.6)		30 (73.2)	9 (22.0)	2 (4.9)	

We applied another three methods for the sensitivity analysis (Supplementary Table 1). The direction of the results is consistent with the primary analysis based on propensity matching.

## Discussion

Our study implied that early active bronchoscopy in patients with PTB can detect TBTB patients with insidious symptoms in a timely manner, thereby reducing the risk of tracheobronchial stenosis. With 12 months of treatment, the symptoms and range of bronchoscopic lesions of patients with non-fibrotic tracheobronchial stenosis were significantly improved in the active group compared with non-active group. However, there were no significant differences for the degree of fibrotic tracheobronchial stenosis and length of stay in the hospital between the two groups. Moreover, the times of interventional therapy and total costs of therapy were significantly higher in the non-active group than that in the active group. In a word, the above hinted that an active bronchoscopy examination was beneficial to early detection of TBTB, thus reducing the proportion of tracheobronchial stenosis and improving prognosis.

Early diagnosis and treatment are key to prevent the transmission of *Mycobacterium tuberculosis*. Bronchoscopy plays an important role in the diagnosis of TBTB, especially in suspicious patients with negative sputum smears and endobronchial diseases (8, 11–13). Since bronchoscopy is not routinely performed in patients with PTB, the incidence of TBTB is uncertain, ranging from 10 to 40%, and even higher (4–7). In a large-scale study, 23.9% of 1,442 patients with PTB were diagnosed with TBTB, the main types being inflammatory infiltration and ulcer necrosis (5). However, in this study, the ratio of patients with TBTB in the active group was higher than that in the literature mentioned above. The main reason may be that every patient diagnosed with PTB in the active group received early and regular bronchoscopy examination, and more hidden TBTB was detected. In recent years, it has been shown that early manifestations of TBTB include mucosal hyperemia, ulceration, and necrosis (2, 3, 7, 9). However, as effective anti-tuberculosis therapy is implemented, most patients gradually improve and return to normal, but a small number of patients may develop tracheobronchial stenosis (14). In our study, early and regular bronchoscopy was performed in the active group, which may have helped to timely identify those patients with TBTB who returned to normal after treatment. This suggests the need for early and regular bronchoscopy in patients with PTB.

The most common complication of TBTB is tracheobronchial stenosis (14, 15). Review of previous literature, the incidence of tracheobronchial stenosis is about 20–60% (3, 5–7). In our study, more than 90% patients with TBTB in the non-active group were combined with airway stenosis, which was significantly higher than active group. The possible reason is that patients with TBTB in the active group were diagnosed early and received timely treatment, which improved the prognosis and lowered the proportion of airway stenosis. But in the non-active group, bronchoscopy was only performed for clinical consultation or at the end of follow-up, which may lead to the development of airway stenosis. Hence, early and timely screening of patients with PTB may improve the prognosis and reduce the proportion of patients with airway stenosis.

With 12 months of treatment, it indicated that patients without definite fibrotic changes in both groups had improvement in bronchoscopic lesions, which was more pronounced in the active group. As predicted, there was no difference in treatment outcomes between the two groups in patients with already formed fibrotic lesions. Thus, it is important to make an early diagnosis of TBTB. When it is clinically suspected, bronchoscopy is recommended to confirm the diagnosis and anti-tuberculosis treatment should be promptly initiated before fibrotic changes are evident.

Now, how to prevent airway stenosis is still unclear. Tata et al. (16) found that the proliferation and migration of myoepithelial cells of submucosal glands can repair airway surface epithelium. Besides, several other studies discovered a variety of fibroblasts in the skin that play different roles in wound repair (17–19). Jiang et al. (18) found that those did not express Engrailed-1 lineage-negative fibroblasts (ENFs) can be activated and transformed into those that expressed Engrailed-1 lineage-positive fibroblasts (EPFs) under the influence of mechanical properties. EPFs were widely present in wounds. Inhibition of this activation of ENFs by the Yes-associated protein pathway almost completely prevents scar formation (19). Therefore, we speculate that early diagnosis and treatment can control airway inflammation in the early stage of TBTB, thereby preventing the occurrence and development of airway fibrosis.

As with all observational studies, the main limitation of our study was uncontrolled confounding factors. At the time of enrollment, clinicians may have counseled symptomatic or serious symptomatic patients to be included in the active group, resulting in selection bias. There might be a potential impact on these findings. Hence, we used PSM to weaken the disequilibrium of intergroup bias (20, 21). In this study, we selected the variables which were related to both the outcome of observation and the processing factors. After matching, it revealed a much higher ratio of TBTB in patients with PTB for undergoing active bronchoscopy examination. Simultaneously, we performed another three methods for the sensitivity analysis to ensure the reliability of these results.

Despite these limitations, our research has some advantages. First, this study is the first observational study to explore the effect of early and regular bronchoscopy on the occurrence and prognosis of TBTB. Second, the study found that early and regular bronchoscopy can detect patients with TBTB in time, so that more effective treatment measures were taken sufficiently in the early stage, thus reducing the proportion of airway stenosis, lowering the risk of disability, and cutting down the consumption of medical resources. Finally, our study suggested that TBTB had a high prevalence in PTB population, and more effective diagnostic and therapeutic measures for TBTB should be explored in future studies.

## Conclusion

Early and regular bronchoscopy examination may be associated with a timely diagnosis and better prognosis of TBTB in patients with PTB. With the popularity of bronchoscopy, clinicians should grasp the indications and contraindications of bronchoscopy, make risk assessment, and maximize the benefits.

## Data Availability Statement

The raw data supporting the conclusions of this article will be made available by the authors, without undue reservation.

## Ethics Statement

The studies involving human participants were reviewed and approved by Medical Ethics Committee of the First Affiliated Hospital of Chongqing Medical University. The patients/participants provided their written informed consent to participate in this study.

## Author Contributions

TH and SG contributed to the conception and design of the study. TH organized the database. TH, RZ, and YinL performed the statistical analysis. TH wrote the first draft of the manuscript. YinL, XW, YC, and XN wrote sections of the manuscript. All authors contributed to manuscript revision, read, and approved the submitted version.

## Funding

This study was funded by the National Science and Technology Major Project of China (2018ZX10302302003).

## Conflict of Interest

The authors declare that the research was conducted in the absence of any commercial or financial relationships that could be construed as a potential conflict of interest.

## Publisher's Note

All claims expressed in this article are solely those of the authors and do not necessarily represent those of their affiliated organizations, or those of the publisher, the editors and the reviewers. Any product that may be evaluated in this article, or claim that may be made by its manufacturer, is not guaranteed or endorsed by the publisher.
