# Supplementary material for: Early and Regular Bronchoscopy Examination on Effect of Diagnosis and Prognosis for Patients With Tracheobronchial Tuberculosis
Source: Front Med (Lausanne). 2022 Feb 15;9:825736. doi: 10.3389/fmed.2022.825736 (PMC8887597; doi:10.3389/fmed.2022.825736)
Supplement: Supplementary file 1 [file Data_Sheet_1.docx]

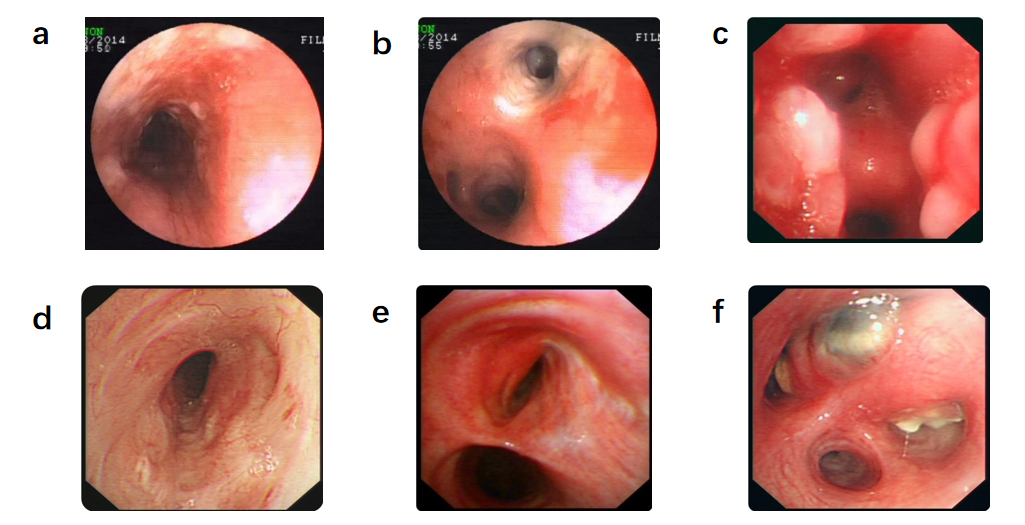


Figure 1. a) Inflammatory infiltration; b) Ulcer necrosis; c) Granulation hyperplasia; d) Cicatricial stenosis; e) Tracheobronchial malacia; f) Lymph fistula

| **Table 1. Prognosis of subtypes of TBTB after a 12-month treatment** | | | | |
| --- | --- | --- | --- | --- |
| Subtypes (%) | Improvement | No Change | Aggravation | P Value |
| Inflammatory infiltration | 28 (87.5) | 3 (9.4) | 1 (3.1) | 0.057 |
| Ulcer necrosis | 31 (75.6) | 6 (14.6) | 4 (9.8) |  |
| Granulation hyperplasia | 8 (88.9) | 1 (11.1) | 0 (0.0) |  |
| Cicatricial stenosis | 60 (76.9) | 16 (20.5) | 2 (2.6) |  |
| Tracheobronchial malacia | 4 (44.4) | 4 (44.4) | 1 (11.1) |  |
| Lymph fistula | 8 (100.0) | 0 (0.0) | 0 (0.0) |  |
